# Dosimetric effects of sectional adjustments of collimator angles on volumetric modulated arc therapy for irregularly-shaped targets

**DOI:** 10.1371/journal.pone.0174924

**Published:** 2017-04-06

**Authors:** Beom Seok Ahn, So-Yeon Park, Jong Min Park, Chang Heon Choi, Minsoo Chun, Jung-in Kim

**Affiliations:** 1 Department of Radiation Oncology, Seoul National University Hospital, Seoul, Republic of Korea; 2 Biomedical Research Institute, Seoul National University College of Medicine, Seoul, Republic of Korea; 3 Institute of Radiation Medicine, Seoul National University Medical Research Center, Seoul, Republic of Korea; 4 Center for Convergence Research on Robotics, Advanced Institutes of Convergence Technology, Suwon, Republic of Korea; North Shore Long Island Jewish Health System, UNITED STATES

## Abstract

**Purpose:**

To calculate an optimal collimator angle at each of sectional arcs in a full-arc volumetric modulated arc therapy (VMAT) plan and evaluate dosimetric quality of these VMAT plans comparing full-arc VMAT plans with a fixed collimator angle.

**Methods:**

Seventeen patients who had irregularly-shaped target in abdominal, head and neck, and chest cases were selected retrospectively. To calculate an optimal collimator angle at each of sectional arcs in VMAT, integrated MLC apertures which could cover all shapes of target determined by beam’s-eye view (BEV) within angular sections were obtained for each VMAT plan. The angular sections were 40°, 60°, 90° and 120°. When the collimator settings were rotated at intervals of 2°, we obtained the optimal collimator angle to minimize area size difference between the integrated MLC aperture and collimator settings with 5 mm-margins to the integrated MLC aperture. The VMAT plans with the optimal collimator angles (Colli-VMAT) were generated in the Eclipse^TM^. For comparison purposes, one full-arc VMAT plans with a fixed collimator angles (Std-VMAT) were generated. The dose-volumetric parameters and total MUs were evaluated.

**Results:**

The mean dose-volumetric parameters for target volume of Colli-VMAT were comparable to Std-VMAT. Colli-VMAT improved sparing of most normal organs but for brain stem, compared to Std-VMAT for all cases. There were decreasing tendencies in mean total MUs with decreasing angular section. The mean total MUs for Colli-VMAT with the angular section of 40° (434 ± 95 MU, 317 ± 81 MU, and 371 ± 43 MU for abdominal, head and neck, and chest cases, respectively) were lower than those for Std-VMAT (654 ± 182 MU, 517 ± 116 MU, and 533 ± 25 MU, respectively).

**Conclusions:**

For an irregularly-shaped target, Colli-VMAT with the angular section of 40° reduced total MUs and improved sparing of normal organs, compared to Std-VMAT.

## Introduction

Volumetric modulated arc therapy (VMAT) have been developed to enable rapid delivery of intensity-modulated photon beams by simultaneous modulations of mechanical parameters including multi-leaf collimator (MLC) positions, gantry rotation speed and dose-rate [1, 2]. Over the past few years, several studies have reported that VMAT plans provide better dose conformity to target tissues and reduce dose to normal tissues with short delivery time, compared to intensity modulated radiation therapy (IMRT) [3–5]. For these reasons, VMAT has been widely adopted for various treatment sites in the clinic [6–12].

For the VMAT planning, the user should determine parameters such as gantry start angle, rotation direction, arc length, gantry spacing, number of arcs, and collimator angles. The collimator angle is a very important parameter to gain a better dosimetric efficiency since it determines the optimization of freedom to shape a desired dose distribution and doses to normal organs could be controlled by blocking the organs properly [13]. Current technology could not rotate the collimator during beam delivery. The optimal choice of the collimator angle has been controversial for the VMAT planning. Otto *et al*. have demonstrated that a collimator angle of 45° has been found to be suitable in most cases [1, 5]. Bortfeld and Webb have tried to do calculations with a collimator angle of 0° for a single-arc IMRT technique [14]. With these studies, Treutwein have assessed several combinations of parameters for VMAT plan optimization and then proved that collimator angle of 45° had better dosimetric quality than a collimator angle of 0° for prostate cancer [15]. In the Eclipse^TM^ (Varian Medical Systems, Palo Alto, CA), they provided the collimator setup before VMAT plan optimization, which included collimator rotations of 10° (350°) and 30° (330°) depending on target size and placement [16].

Furthermore, several studies have found that dynamic collimator angles during VMAT had the potential to improve the dosimetric efficiency. Zhang *et al*. have developed a collimator trajectory optimization algorithm for paraspinal stereotactic body radiation therapy (SBRT) applying principal component analysis (PCA). The principal component calculated from beam’s-eye view (BEV) of the spinal cord could express the long axis of the cord and then determine the proper MLC direction. They have evaluated the potential for dosimetric improvements, compared to VMAT with a fixed collimator angle [13]. Boer *et al*. have performed collimator and gantry angle adjustments during VMAT delivery in order to consider left-right prostate rotations. With this technique, they have found that left-right prostate rotations were up to 15° and then could be corrected [17].

**Fig 1:**
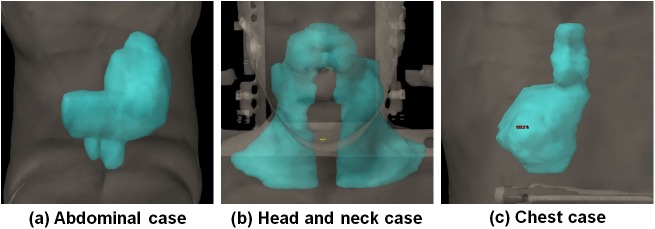
Irregularly-shaped target volumes for abdomen (a), head and neck (b), and chest cases (c).

In cases of the abdomen, head and neck, and chest region as well as the spinal cord and prostate region examined by previous studies [13, 17], the targets are large and irregularly shaped as shown in Fig 1. As the gantry rotates, BEVs of these large and irregularly-shaped targets at each control point (CP) are changed dramatically. VMAT plans with a fixed collimator angle do not cover all BEVs of these targets, which make target coverage and target dose uniformity, and dose delivered to peripheral organs uncontrollable. A better understanding of the effect of the collimator angles on VMAT planning is needed to support a high-quality radiation therapy.

The purpose of this study was to calculate the optimal collimator angles at each sectional arc in a full-arc VMAT for the large irregularly-shaped target in cases of the abdomen, head and neck, and chest and then evaluate the dosimetric quality of the VMAT plans with the optimal collimator angles (Colli-VMAT), compared to those of the VMAT plans with a fixed collimator angle (Std-VMAT).

## Materials and methods

### Patient selection

Eight patients for abdominal cases, six patients for head and neck cases, and three patients for chest cases who had an irregularly-shaped target were chosen retrospectively. It was approved by the institutional review board of Seoul National University College of Medicine/Seoul National University Hospital (IRB No.1509-124-706) for this study. Table 1 lists the patient characteristics for abdomen, head and neck, and chest cases, consisting of the target volume, treatment site, and prescription dose. The average of the target volume were 664.2 cc, 286.2 cc, and 385.0 cc for abdomen, head and neck, and chest cases, respectively. Prescription doses were ranged from 30 Gy to 54 Gy according to the treatment site.

**Table 1:** Patient characteristics.

	Patient ID	Target volume (cc)	Treatment site	Prescription dose (Gy)
Abdomen	1	684.2	Stomach	30.6
	2	770.8	Stomach	45.0
	3	923.3	Stomach	30.6
	4	383.2	Stomach	45.0
	5	342.0	Liver	36.0
	6	392.4	Gallbladder	50.0
	7	1558.4	Lymphoblastic lymphoma	30.0
	8	258.9	Right retroperitoneum	45.0
Head and neck	1	269.8	Tongue	48.0
	2	217.2	Neck	56.0
	3	314.1	Nasopharynx	54.0
	4	443.2	Tonsil	48.0
	5	209.6	Neck	47.6
	6	263.4	Tonsil	47.6
Chest	1	424.2	Lung	44.0
	2	337.0	Lung	45.0
	3	393.7	Lung	54.0

### Determination of optimal collimator angles

In this study, we used a commercial treatment planning system, Eclipse^TM^. In order to obtain MLC apertures for the irregularly-shaped target and then generate integrated MLC apertures within sectional arcs, the conformal arc plan first of all was generated for each patient using one full-arc with a collimator angle of 0°. The number of CPs for the conformal arc plan was 500, which is the maximum value with 0.72°/CP spacing. The generated MLC apertures encompassed the target volume over each CP. Each conformal arc plan was exported in DICOM-RT format files from the Eclipse^TM^. Using an in-house program written in MATLAB (ver.8.1, Mathworks, Inc., Natick, MA), MLC positions for each CP were obtained, which represented the outline of the target. Since the available minimum-gantry angle for VMAT plans is 40° in the Eclipse^TM^, the angular sections used in our study were 40°, 60°, 90°, and 120°, respectively. For one full-arc of 360°, VMAT plans had sectional arcs (partial arc field) of 9, 6, 4, and 3 according to the angular sections of 40°, 60°, 90° and 120°, respectively. For each of sectional arcs in VMAT plan, integrated MLC apertures which selected MLC positions having the largest gap within the sectional arc were generated. The integrated MLC apertures could cover all shapes of target within the sectional arc. When the collimator settings were rotated ranging from 0° to 90° at intervals of 2°, we obtained the optimal collimator angles that minimized an area size difference between the integrated MLC aperture and the collimator settings with 5 mm-margins to the integrated MLC aperture. For better illustration purpose, S1 Appendix provides the more detailed procedure to calculate the optimal collimator angles using diagrams. This process was repeated to calculate the optimal collimator angle for each sectional arc. The VMAT plans with the optimal collimator angles (Colli-VMAT) were generated in the Eclipse^TM^. For comparison purposes, one full-arc VMAT plans with a fixed collimator angle (Std-VMAT) which had an angular section of 360° were generated. The fixed collimator angle for Std-VMAT was determined using the same protocol for calculating the optimal collimator angle of Colli-VMAT.

### Treatment planning

Before VMAT plan optimization, some of calculated optimal collimator angles for sectional arcs were re-determined by adding 90° rotation to those calculated optimal collimator angles. If the collimator angle added to 90° rotation had the potential for reducing the travel distances of the MLCs from the MLC carriage for modulating intensity of photon beams, the collimator angles added to 90° were re-selected as the optimal collimator angles. All VMAT plans were optimized with the progressive resolution optimizer 3 (PRO3, ver. 10, Varian Medical Systems, Palo Alto, CA) for 6 MV photon beam. To improve the dosimetric quality in VMAT plans, all VMAT plans were re-optimized using the current dose distribution as a reference for re-optimization. The dose distributions were calculated by using the anisotropic analytic algorithm (AAA, ver. 10, Varian Medical Systems, Palo Alto, CA) with a calculation grid of 1 mm which is for removing the calculation grid dependency. All plans were normalized so that 98% of the prescription dose covered 98% of the target volume.

### Dosimetric evaluation and delivery efficiency

The dosimetric quality with respect to target coverage and dose received by normal organs was assessed by analysing dose-volume histogram (DVH) data. For the target volume, the evaluated dose-volumetric parameters were the mean dose, maximum dose, minimum dose, dose received by at least 97% volume of the target volume (D_97%_), the percent volume of the target volume irradiated by at least 95% of the prescription dose (V_95%_), V_97%_, conformity index suggested by Paddick (CI_Paddick_), homogeneity index (HI), and gradient measure (GM). The CI_Paddick_, HI, and GM are defined as follows [18–22]:

$${CI}_{Paddick}=\frac{{TV}_{prescription\;dose}}{TV}\times \frac{{TV}_{prescription\;dose}}{V_{prescription\;dose}}$$ (1)

$$HI=\frac{D_{2\%}-D_{98\%}}{D_{50\%}}$$ (2)

$$GM=R_{50\%\;of\;prescription\;dose}-R_{prescription\;dose}$$ (3)

where, the *TV*_*prescription dose*_ is the target volume irradiated by the prescription dose, *TV* is the target volume, *V*_*prescription dose*_ is the volume of the prescription dose, and *R*_*x*_ is the sphere radius of which the volume is the same as the volume of isodose of *x*.

For the normal organ, D_5%_, maximum dose, and mean dose of the spinal cord, V_30 Gy_ and mean dose of pancreas as well as liver and esophagus, V_35 Gy_ and mean dose of the duodenum as well as stomach, maximum dose and mean dose of the brain stem, maximum dose of right lens as well as left lens, right parotid and left parotid, V_34 Gy_ and mean dose of the heart, and V_20 Gy_, V_5 Gy_, and mean dose of the right lung as well as left lung were calculated.

Mean total MUs were compared and mean total MU reduction was also calculated to evaluate the relative delivery efficiency of each VMAT plan. The total MU reduction are defined as follows:

$$Total\;MU\;reduction\;(\%)=\frac{{MU}_{Colli-VMAT}-{MU}_{Std-VMAT}}{{MU}_{Std-VMAT}}\times 100$$ (4)

where, *MU*_*colli-VMAT*_ and *MU*_*Std-VMAT*_ are total MUs for the Colli-VMAT and Std-VMAT, respectively.

## Results

### Optimal collimator angle

**Fig 2:**
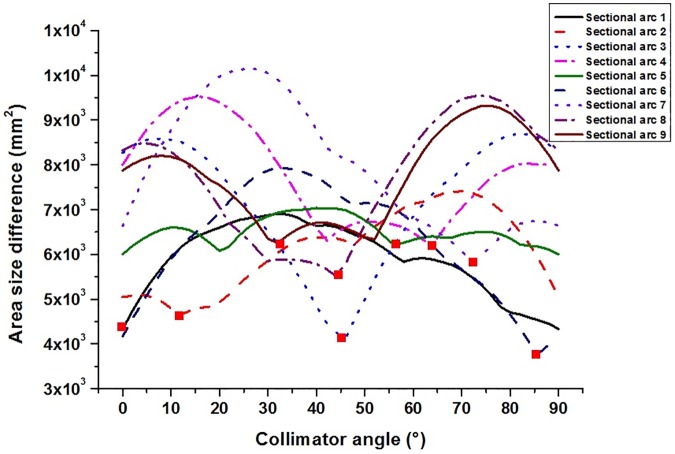
An example of area size difference between the integrated multi-leaf collimator (MLC) aperture and collimator settings with 5 mm-margins to the integrated MLC aperture with respect to collimator angles in the case of an angular section of 40°. Collimator angles were rotated at intervals of 2° ranging from 0° to 90°. Red (square) points for each sectional arc were minimum area size differences.

For 9 sectional arcs with an angular section of 40°, an example of the area size differences with regard to collimator angles in the case of the abdominal region are shown in Fig 2. When a collimator is rotated from 0° to 90°, there are various tendencies which could not be predictable for each sectional arc but there should be minimum area size differences represented by the red (square) point in Fig 2. An example of optimal collimator angles at each of sectional arcs in VMAT for abdomen cases is shown in Table 2. The optimal collimator angles have the values that does not overlap at least two for more collimator angles successively. The optimal collimator angles of 348°, 312°, 296°, and 304° for sectional arcs with angular sections of 40°, 316° and 298° for sectional arcs with angular sections of 60°, and 300° for sectional arcs with angular sections of 90° and 120° are re-selected in order to decrease the burden of the MLC control for modulating intensity of photon beam. The initial collimator angles calculated from the minimum area size differences were 12°, 48°, 64°, and 56° for sectional arcs with angular sections of 40°, 44° and 62° for sectional arcs with angular sections of 60°, and 60° for sectional arcs with angular sections of 90° and 120°. The collimator angle of the Std-VMAT plan for all cases has the value of 0°. We recognized similar behaviours in other VMAT plans. The integrated MLC apertures considering all shapes of target volume had broad and isotropic shapes. When the collimator angle was 0°, the area size difference was minimum for the Std-VMAT plan.

**Table 2:** The values of optimal collimator angles for each of sectional arcs for abdominal case.

	Sectional arc								
	1	2	3	4	5	6	7	8	9
Colli-VMAT (40°)	0°	348°	312°	296°	304°	4°	18°	48°	58°
Colli-VMAT (60°)	0°	316°	298°	2°	90°	58°	-	-	-
Colli-VMAT (90°)	0°	300°	6°	45°	-	-	-	-	-
Colli-VMAT (120°)	0°	0°	38°	-	-	-	-	-	-
Std-VMAT (360°)	0°	-	-	-	-	-	-	-	-

### Dosimetric evaluation and delivery efficiency

**Fig 3:**
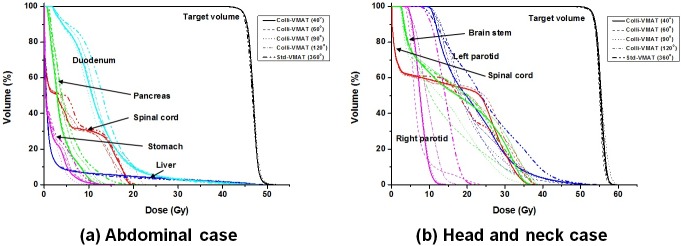
**Examples of dose-volume histograms of target volumes and normal organs for abdomen cases (a), head and neck cases (b).** The DVHs of Colli-VMAT plans with an angular section of 40° (Colli-VMAT (40°)) and Std-VMAT plans (Std-VMAT (360°)) are plotted with bold lines (solid and dashed) while those of Colli-VMAT plans with angular sections of 60°, 90°, and 120° (Colli-VMAT (60°), Colli-VMAT (90°), and Colli-VMAT (120°)) are plotted with dotted, dash-dotted, and dash-dot-dotted lines, respectively.

The dose-volumetric parameters of the Std-VMAT plan (with an angular section of 360°) and Colli-VMAT plans with angular sections of 40°, 60°, 90°, and 120° in abdomen cases are shown in Table 3. The patient-mean values of D_97%_, V_97%_, maximum dose, mean dose, CI_Paddick_, HI, and GM for the target volume were similar regardless of the value of angular sections, while those of V_95%_, and minimum doses were decreased as the values of angular sections were decreased. For normal organs in abdomen cases, the patient- mean values of D_5%_, maximum dose, and mean dose for a spinal cord, V_30 Gy_, and mean dose for a pancreas, and V_30 Gy_ for a liver had decreasing tendencies with decreasing values of angular sections. Most of the values of dose-volumetric parameters for normal organs were lower when Colli-VMAT plans were used, compared to the Std-VMAT plan. The example of DVH for abdominal case is shown in Fig 3(A). The dose-volumetric parameters of the Std-VMAT plan and Colli-VMAT plans with angular sections of 40°, 60°, 90°, and 120° in cases of the head and neck are shown in Table 4. The values of dose-volumetric parameters for the target volume and most normal organs did not show noticeable tendencies regardless of the value of angular sections, except that the values of mean dose for target volume, and maximum dose for brain stem had increasing tendencies and those of minimum dose for the target volume had decreasing tendencies as the value of angular sections were decreased. Among the six patients in head and neck case, there were advantages of coverage of the target volume and normal organ sparing when Colli-VMAT plans with angular sections of 40°and 60° were used, shown in Fig 3(B). The dose-volumetric parameters of the Std-VMAT plan and Colli-VMAT plans with angular sections of 40°, 60°, 90°, and 120° in chest case are shown in Table 5. The values of dose-volumetric parameter for the target volume did not show noticeable tendencies regardless of the value of angular sections. The values of D_5%_, maximum dose, and mean dose for spinal cord, V_34 Gy_ for heart had decreasing tendencies when the value of angular sections became smaller. The values of V_20 Gy_, V_5 Gy_, and mean dose for right lung, and V_5 Gy_ for left lung obtained from every Colli-VMAT plans were lower, compare to those obtained from the Std-VMAT plan. Overall, the mean dose-volumetric parameters for the target volume of Colli-VMAT plans were comparable to Std-VMAT plans and also Colli-VMAT plans improved sparing of the most normal organs compared to Std-VMAT plans for all cases.

**Table 3:** Average dose-volumetric parameters of abdominal cases.

		Colli-VMAT (40°)	Colli-VMAT (60°)	Colli-VMAT (90°)	Colli-VMAT (120°)	Std-VMAT (360°)
Target volume	D_97%_ (Gy)	38.6 ± 8.0	38.5 ± 8.0	38.6 ± 7.9	38.5 ± 8.0	38.5 ± 8.0
	V_95%_ (%)	99.4 ± 0.2	99.5 ± 0.2	99.5 ± 0.1	99.5 ± 0.3	99.6 ± 0.1
	V_97%_ (%)	98.7 ± 0.1	98.8 ± 0.2	98.9 ± 0.2	98.7 ± 0.5	98.9 ± 0.1
	Minimum dose (Gy)	28.2 ± 7.3	28.7 ± 6.7	29.6 ± 8.7	29.6 ± 7.5	29.6 ± 7.5
	Maximum dose (Gy)	45.1 ± 9.4	44.9 ± 9.5	45.8 ± 9.5	44.8 ± 9.6	45.2 ± 10.0
	Mean dose (Gy)	41.0 ± 8.4	40.6 ± 8.3	40.9 ± 8.3	40.4 ± 8.4	40.5 ± 8.3
	CI_paddick_	0.8 ± 0.1	0.8 ± 0.1	0.8 ± 0.1	0.8 ± 0.1	0.8 ± 0.1
	HI	1.1 ± 0.0	1.1 ± 0.0	1.1 ± 0.0	1.1 ± 0.0	1.1 ± 0.0
	GM (cm)	1.4 ± 0.1	1.4 ± 0.2	1.4 ± 0.1	1.4 ± 0.1	1.5 ± 0.1
Spinal cord	D_5%_ (Gy)	15.5 ± 6.5	15.4 ± 6.6	16.3 ± 7.3	17.8 ± 5.9	19.9 ± 5.3
	Maximum dose (Gy)	17.5 ± 7.2	17.3 ± 7.1	18.8 ± 7.9	20.0 ± 6.3	22.9 ± 6.0
	Mean dose (Gy)	6.3 ± 3.2	6.2 ± 3.1	6.3 ± 3.2	7.0 ± 3.0	7.8 ± 2.9
Pancreas	V_30 Gy_ (%)	42.3 ± 21.0	41.0 ± 22.0	43.7 ± 20.0	45.5 ± 18.4	46.0 ± 17.4
	Mean dose (Gy)	23.6 ± 14.9	22.9 ± 14.5	23.3 ± 14.9	23.9 ± 14.7	24.6 ± 14.7
Liver	V_30 Gy_ (%)	7.3 ± 7.6	6.9 ± 7.0	8.2 ± 6.3	7.5 ± 7.6	7.5 ± 8.0
	Mean dose (Gy)	10.9 ± 6.5	10.6 ± 6.2	11.0 ± 6.3	10.9 ± 6.5	10.9 ± 6.6
Duodenum	V_35 Gy_ (%)	7.0 ± 14.4	7.0 ± 14.1	8.6 ± 16.1	6.8 ± 14.1	6.4 ± 13.4
	Mean dose (Gy)	15.3 ± 10.7	15.0 ± 10.6	14.9 ± 10.8	15.4 ± 10.6	16.0 ± 10.2
Stomach	V_35 Gy_ (%)	17.3 ± 44.3	17.0 ± 44.4	17.3 ± 44.2	17.0 ± 44.4	16.9 ± 44.5
	Mean dose (Gy)	17.7 ± 19.0	17.4 ± 18.3	17.4 ± 18.7	17.6 ± 18.4	17.4 ± 18.3

Abbreviations: D_n%_ dose received by at least n% volume of the target volume, V_n%_ the percent volume of a structure irradiated by at least n% of the prescription dose, CI conformity index proposed by Paddick *et*. *al*., HI homogeneity indx, GM gradient measure, Colli-VMAT (n°) volumetric modulated arc therapy plan with the optimal collimator angles at angular sections n°, Std-VMAT (360°) volumetric modulated arc therapy plan with a fixed collimator angle at an angular section 360°

**Table 4:** Average dose-volumetric parameters of head and neck cases.

		Colli-VMAT (40°)	Colli-VMAT (60°)	Colli-VMAT (90°)	Colli-VMAT (120°)	Std-VMAT (360°)
Target volume	D_97%_ (Gy)	49.9 ± 3.6	49.7 ± 3.6	49.5 ± 3.9	49.7 ± 3.7	49.7 ± 3.7
	V_95%_ (%)	99.3 ± 0.4	99.4 ± 0.3	99.2 ± 0.7	99.5 ± 0.2	99.4 ± 0.2
	V_97%_ (%)	98.6 ± 0.2	98.7 ± 0.3	98.4 ± 0.9	98.7 ± 0.2	98.7 ± 0.2
	Minimum dose (Gy)	31.3 ± 8.3	33.7 ± 7.0	34.0 ± 6.0	31.9 ± 5.4	36.0 ± 5.3
	Maximum dose (Gy)	59.2 ± 4.5	58.7 ± 4.2	59.2 ± 4.2	59.0 ± 4.1	60.3 ± 4.7
	Mean dose (Gy)	53.4 ± 3.7	52.9 ± 3.6	52.7 ± 4.0	52.7 ± 3.5	52.6 ± 3.7
	CI_paddick_	0.6 ± 0.1	0.6 ± 0.1	0.6 ± 0.1	0.6 ± 0.1	0.6 ± 0.1
	HI	1.1 ± 0.0	1.1 ± 0.0	1.1 ± 0.0	1.1 ± 0.0	1.1 ± 0.0
	GM (cm)	1.1 ± 0.2	1.1 ± 0.2	1.1 ± 0.1	1.1 ± 0.1	1.1 ± 0.1
Spinal cord	D_5%_ (Gy)	35.1 ± 14.6	34.4 ± 14.3	33.8 ± 10.7	34.8 ± 12.6	34.8 ± 11.5
	Maximum dose (Gy)	37.9 ± 14.1	37.1 ± 14.2	37.6 ± 10.0	37.4 ± 13.0	37.6 ± 0.3
	Mean dose (Gy)	18.6 ± 9.8	18.0 ± 9.3	17.8 ± 7.8	18.0 ± 8.7	17.5 ± 8.1
Brain stem	Maximum dose (Gy)	16.2 ± 13.8	15.0 ± 13.8	14.2 ± 13.9	15.2 ± 14.1	14.9 ± 14.3
	Mean dose (Gy)	5.2 ± 6.8	4.4 ± 5.2	4.6 ± 5.4	5.4 ± 7.2	5.1 ± 6.9
Right lens	Maximum dose (Gy)	0.6 ± 0.5	0.7 ± 0.5	0.6 ± 0.5	0.6 ± 0.5	0.6 ± 0.5
Left lens	Maximum dose (Gy)	0.7 ± 0.6	0.6 ± 0.6	0.6 ± 0.6	0.6 ± 0.6	0.6 ± 0.6
Right parotid	Maximum dose (Gy)	18.5 ± 12.9	17.6 ± 12.5	16.9 ± 11.1	17.6 ± 11.2	18.0 ± 10.3
Left parotid	Maximum dose (Gy)	12.8 ± 10.7	12.4 ± 10.5	11.7 ± 9.6	12.8 ± 10.1	13.2 ± 10.2

Abbreviations: D_n%_ dose received by at least n% volume of the target volume, V_n%_ the percent volume of a structure irradiated by at least n% of the prescription dose, CI conformity index proposed by Paddick *et*. *al*., HI homogeneity indx, GM gradient measure, Colli-VMAT (n°) volumetric modulated arc therapy plan with the optimal collimator angles at angular sections n°, Std-VMAT (360°) volumetric modulated arc therapy plan with a fixed collimator angle at an angular section 360°

**Table 5:** Average dose-volumetric parameters of chest cases.

		Colli-VMAT (40°)	Colli-VMAT (60°)	Colli-VMAT (90°)	Colli-VMAT (120°)	Std-VMAT (360°)
Target volume	D_97%_ (Gy)	47.1 ± 5.5	47.1 ± 5.6	47.2 ± 5.6	46.8 ± 5.8	47.1 ± 5.5
	V_95%_ (%)	99.5 ± 0.1	99.6 ± 0.1	99.6 ± 0.2	99.3 ± 0.4	99.6 ± 0.2
	V_97%_ (%)	98.8 ± 0.1	98.8 ± 0.1	98.8 ± 0.2	98.2 ± 1.1	98.8 ± 0.2
	Minimum dose (Gy)	34.3 ± 8.2	34.0 ± 8.2	35.0 ± 8.0	36.1 ± 8.6	35.1 ± 7.6
	Maximum dose (Gy)	54.9 ± 6.9	55.2 ± 6.5	56.4 ± 6.1	54.4 ± 6.8	55.2 ± 7.2
	Mean dose (Gy)	50.2 ± 6.1	45.0 ± 6.2	50.2 ± 6.2	49.5 ± 6.5	49.9 ± 6.5
	CI_paddick_	0.8 ± 0.0	0.8 ± 0.0	0.8 ± 0.0	0.8 ± 0.0	0.8 ± 0.0
	HI	1.1 ± 0.0	1.1 ± 0.0	1.1 ± 0.0	1.1 ± 0.0	1.1 ± 0.0
	GM (cm)	1.2 ± 0.1	1.2 ± 0.1	1.3 ± 0.1	1.3 ± 0.1	1.3 ± 0.2
Spinal cord	D_5%_ (Gy)	22.4 ± 5.0	23.0 ± 4.4	24.6 ± 4.5	24.4 ± 3.6	26.6 ± 2.8
	Maximum dose (Gy)	24.5 ± 5.3	25.4 ± 4.9	27.7 ± 4.5	27.0 ± 3.9	29.4 ± 3.5
	Mean dose (Gy)	7.1 ± 1.4	7.4 ± 1.5	8.0 ± 1.6	7.7 ± 1.5	8.3 ± 1.4
Esophagus	V_30 Gy_ (%)	44.8 ± 13.1	42.3 ± 14.5	44.4 ± 13.5	41.7 ± 14.1	44.7 ± 14.9
	Mean dose (Gy)	23.3 ± 7.6	22.9 ± 7.6	23.6 ± 8.0	23.2 ± 8.3	23.9 ± 8.5
Heart	V_34 Gy_ (%)	6.5 ± 1.9	6.8 ± 1.2	6.4 ± 1.7	6.7 ± 0.9	7.1 ± 1.7
	Mean dose (Gy)	12.5 ± 2.6	12.4 ± 2.3	11.2 ± 1.6	12.7 ± 2.8	12.8 ± 2.1
Right lung	V_20 Gy_ (%)	38.0 ± 15.6	36.5 ± 13.4	39.8 ± 11.1	37.7 ± 14.5	42.6 ± 10.4
	V_5 Gy_ (%)	89.9 ± 6.5	89.7 ± 5.5	89.1 ± 6.2	90.5 ± 6.9	90.9 ± 6.4
	Mean dose (Gy)	19.3 ± 4.5	19.1 ± 4.0	19.4 ± 3.6	19.4 ± 4.1	20.3 ± 3.8
Left lung	V_20 Gy_ (%)	6.4 ± 8.3	6.6 ± 6.8	5.8 ± 6.8	7.4 ± 9.6	6.8 ± 8.0
	V_5 Gy_ (%)	67.0 ± 12.4	66.6 ± 11.6	64.3 ± 11.0	67.7 ± 11.3	68.5 ± 13.6
	Mean dose (Gy)	9.1 ± 3.1	9.2 ± 3.0	8.7 ± 2.9	9.3 ± 3.1	9.2 ± 3.1

Abbreviations: D_n%_ dose received by at least n% volume of the target volume, V_n%_ the percent volume of a structure irradiated by at least n% of the prescription dose, CI conformity index proposed by Paddick *et*. *al*., HI homogeneity indx, GM gradient measure, Colli-VMAT (n°) volumetric modulated arc therapy plan with the optimal collimator angles at angular sections n°, Std-VMAT (360°) volumetric modulated arc therapy plan with a fixed collimator angle at an angular section 360°

**Fig 4:**
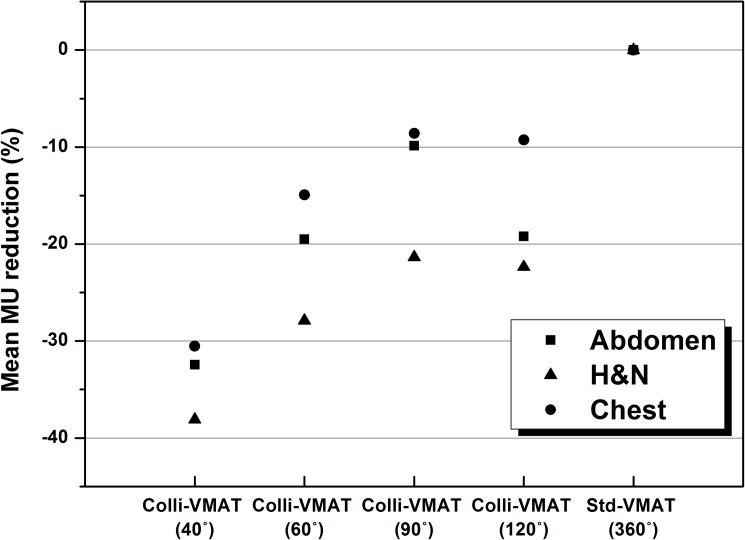
The average values of total MU reduction with respect to Colli-VMAT plans with angular sections of 40°, 60°, 90°, and 120° (Colli-VMAT (40°), Colli-VMAT (60°), Colli-VMAT (90°), and Colli-VMAT (120°)), and Std-VMAT plan (Std-VMAT (360°)) for abdomen, head and neck, and chest cases.

When Colli-VMAT plans with an angular section of 40° were used, mean total MUs (434 ± 95 MU, 317 ± 81 MU, and 371 ± 43 MU for abdominal, head and neck, chest cases, respectively) were lowest as listed in Table 6. For comparison, mean total MUs for the Std-VMAT plan were 654 ± 182 MU, 517 ± 116 MU, and 533 ± 25 MU, respectively. Mean values of total MU reductions for all cases with respect to the value of angular sections are plotted in Fig 4. There were deceasing tendencies in mean total MUs as the value of angular sections was decreased. For Colli-VMAT plans with angular section of 40°, the mean values of total MU reductions were -32.5%, -38.1%, and -31.5% for abdomen, head and neck, chest cases, respectively. Large amount of reduction of total MUs shows improvement on delivery efficiency and reduction of scatter and leakage radiation to patients in VMAT.

**Table 6:** Mean total MUs for abdominal, head and neck, and chest cases.

	Colli-VMAT (40°)	Colli-VMAT (60°)	Colli-VMAT (90°)	Colli-VMAT (120°)	Std-VMAT (360°)
Abdomen	434 ± 95	515 ± 105	576 ± 125	518 ± 107	654 ± 182
Head and neck	317 ± 81	374 ± 115	406 ± 96	401 ± 106	517 ± 116
Chest	371 ± 43	453 ± 20	488 ± 50	484 ± 30	533 ± 25

Abbreviations: Colli-VMAT (n°) volumetric modulated arc therapy plan with the optimal collimator angles at angular sections n°, Std-VMAT (360°) volumetric modulated arc therapy plan with a fixed collimator angle at an angular section 360°

## Discussion

In this study, we calculated the optimal collimator angles for each of sectional arcs in VMAT for a large and irregularly-shaped target to improve the plan quality. DICOM-RT format files of the conformal arc plan generated from the Eclipse^TM^ were used to simply obtain the MLC apertures of the target determined by MLCs within certain angular sections. The results of Colli-VMAT plans with smaller angular sections demonstrated that the coverage of the target volume was comparable and sparing of normal organs could be improved, compared with those of the Std-VMAT plan. Also, there was a highly significant reduction in total MUs with comparable dosimetric quality for Colli-VMAT plans with smaller angular sections. The results of the present study show the similarity to those of the earlier study. Zhang *et al*. reported that VMAT plans with a collimator trajectory improved the target coverage and cord sparing of paraspinal SBRT plans compared to VMAT plan with a fixed collimator angle and IMRT plan. The collimator trajectory provided an additional degree of freedom for VMAT plan optimization [13]. Holtzer *et al*.[23] and Boer *et al*.[17] showed that there was improvement on the effect of the dynamic collimator angle on the dosimetric quality to correct roll and pitch errors in cases of the head and neck, and prostate, respectively. They have demonstrated that this technique could improve planning target volume (PTV) coverage and decrease maximum dose to normal organs. Furthermore, dynamic adjustments of the collimator angle during VMAT is the most ideal way to improve the delivery efficiency and dosimetric quality of VMAT plans.

For the optimal collimator angles within small angular section, area size differences between the integrated MLC aperture and collimator settings was minimum and total MUs of VMAT were decreased dramatically. The minimum area size difference means that the MLC area exposed by photon beams was smaller. It was demonstrated that the portion of scatter and leakage radiation to patients could be decreased due to these effects for treatment. Several studies have demonstrated that the risk of the secondary cancer to patients may be increased by scatter and leakage doses to patients after introducing IMRT and VMAT techniques which use high MUs [24–27]. Therefore, it was important to reduce dose from scatter and leakage radiation for treatment. By minimizing the area size differences and total MUs for our study, the clinical advantage of reduction of secondary cancer risk as well as comparable dosimetric quality of VMAT could be expected [24–27].

As mentioned previously, the minimum angular section of the gantry angle for VMAT plans is 40° in the Eclipse^TM^. If the minimum angular section could be decreased in the Eclipse^TM^, the effect of the optimal collimator angle suggested in this study on the dosimetric quality could be further improved.

When starting our study, 28 patents who had only one large and irregularly-shaped target for abdominal, head and neck, and chest cases was chosen, retrospectively. In the process of calculation of optimal collimator angles for each of sectional arcs, the values of optimal collimator angles may be repeated successively and the target was not considered to be irregular. For that reason, 11 patients who had repeated optimal collimator angles in succession were excluded from our study to show the dramatic effects of optimal collimator angles in VMAT. There is a limitation associated with statistical analysis. It is not appropriate to do statistical analysis because of the small number of samples. Further study using the large number of samples the will be necessary to show statistical analysis.

**Fig 5:**
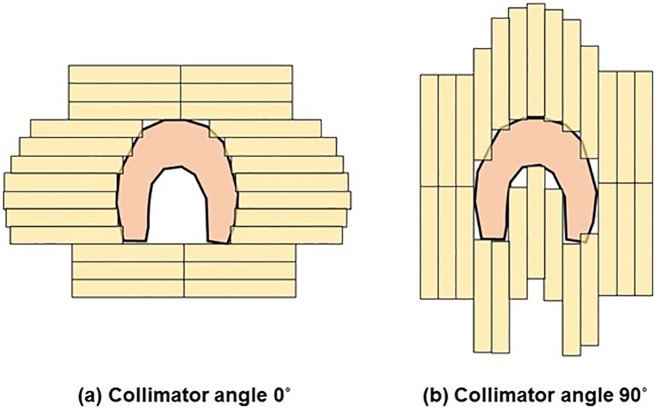
Beam’s-eye view (BEV) of the target volume which has a concave shape fitted by multi-leaf collimators (MLCs) at collimator angles of 0° (a) and 90° (b).

To generate the conformal arc plan for each patient, we used the same collimator angle of 0° for all cases. This fixed collimator angle had limitations which could not fully represent the shape of the target defined by MLCs, as shown in Fig 5. If the targets had concave shapes like head and neck cases or multi-target volumes like brain cases, a fixed collimator angle of 0° used in our study was not appropriate. The results of dose-volumetric parameters for head and neck cases listed in Table 3 showed relatively small improvement in coverage of target volumes and sparing of normal organs when using Colli-VMAT plans, compared to other cases. Further study using various collimator angles of the conformal arc plan in order to fully express the shape of the target will be performed in the future.

## Conclusions

We obtained the optimal collimator angles for each of sectional arcs in VMAT plans to cover the large and irregularly-shaped target effectively. In the case of abdominal, head and neck, and chest cases, Colli-VMAT plans with an angular section of 40° reduced total MUs that can shorten the treatment time and improved sparing of normal tissues, compared to other Colli-VMAT plans with angular sections of 60°, 90°, 120°, and the Std-VMAT plan. By using DICOM-RT format files of the conformal arc plan, the optimal collimator angles could be obtained easily and then Colli-VMAT plans with an angular sections of 40° could be adopted in the clinic for an irregularly-shaped target.

## Supporting information

S1 Appendix Detailed procedure to calculate the optimal collmator angles by detemining the area size difference. (PDF)
