# Supplementary material for: Dosimetric effects of sectional adjustments of collimator angles on volumetric modulated arc therapy for irregularly-shaped targets
Source: PLoS One. 2017 Apr 6;12(4):e0174924. doi: 10.1371/journal.pone.0174924 (PMC5383152; doi:10.1371/journal.pone.0174924)
Supplement: S1 Appendix — (PDF) [file pone.0174924.s001.pdf]

Supporting materials

Determination of the area size difference

1. Acquisition of multi-leaf collimator (MLC) positions, gantry angles and monitor units (MUs) for all control points from DICOM RT plan file.

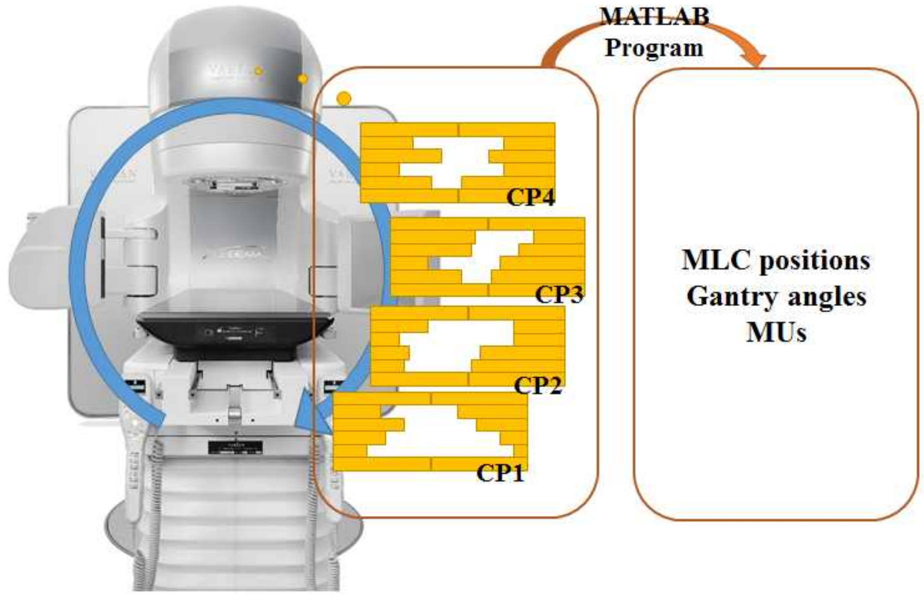

Figure 1. Acquisition of modulating parameters from DICOM RT plan file

2. Generation of the integrated MLC aperture for certain angular section

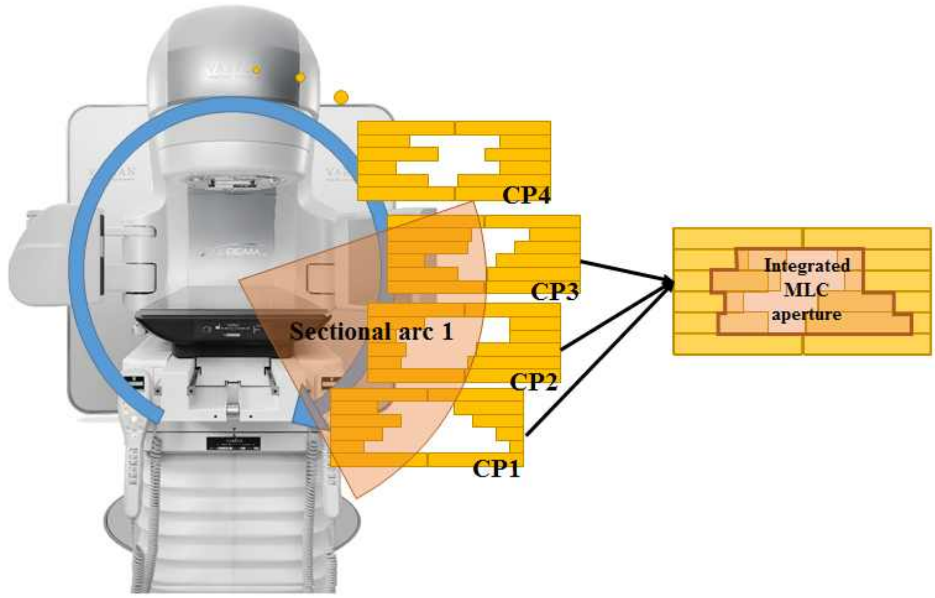

Figure 2. Integrated MLC aperture for certain angular section

3. Collimator settings with 5 mm-margins to the integrated MLC aperture

3.1. Largest gap = Maximum distance between the tip of leftmost left leaf and the tip of the rightmost right leaf of the MLC within the sectional arc

3.2. Integrated MLC aperture = MLC aperture defined by MLC positions having the largest gap within the sectional arc

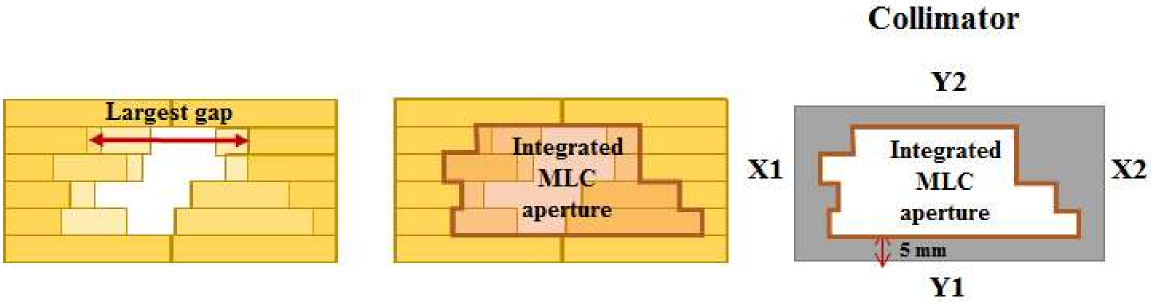

Figure 3. Fitting the collimator size to integrated MLC aperture

4. Collimator rotation to calculate the area size difference

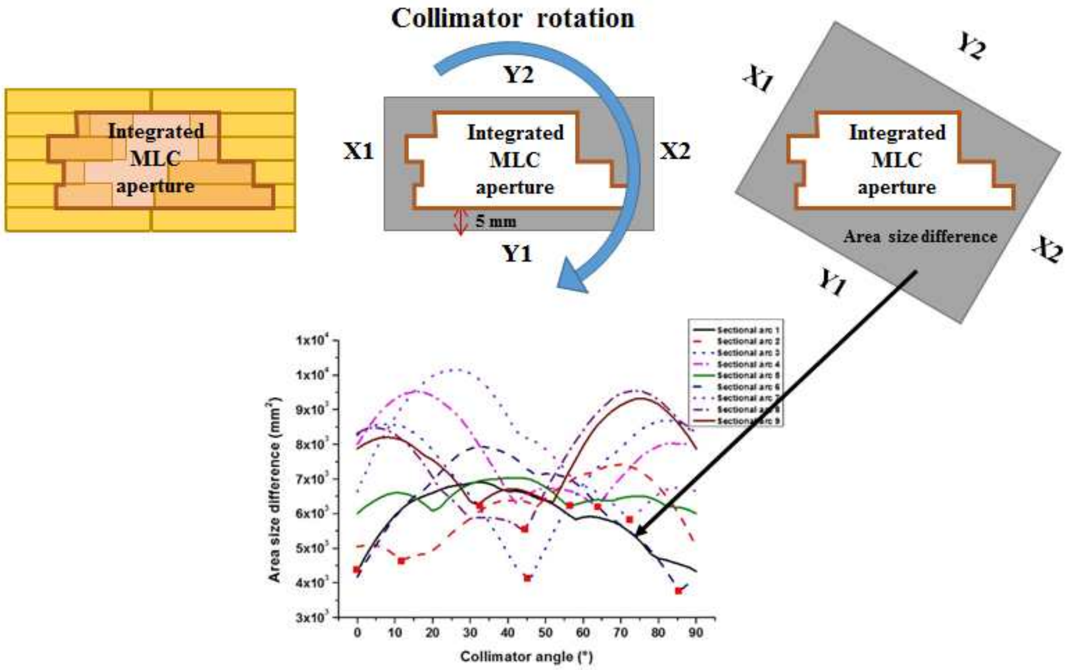

Figure 4. Calculation of the area size difference by rotating collimator settings

5. Find the optimal collimator angle minimizing the area size difference between integrated MLC aperture and the collimator settings with rotation interval of 2˚
